# Whole-genome resequencing reveals the origin of tea in Lincang

**DOI:** 10.3389/fpls.2022.984422

**Published:** 2022-09-15

**Authors:** Yahui Lei, Ling Yang, Shengchang Duan, Siqi Ning, Dawei Li, Zijun Wang, Guisheng Xiang, Ling Yang, Chunping Wang, Shiyu Zhang, Shuangyan Zhang, Shuang Ye, Ling Kui, Pratiksha Singh, Jun Sheng, Yang Dong

**Affiliations:** ^1^College of Food Science and Technology, Yunnan Agricultural University, Kunming, China; ^2^Nowbio Biotechnology Company, Kunming, China; ^3^Experimental Middle School of Yunnan Normal University, Kunming, China; ^4^Yunnan Agricultural University Applied Genomics Technology Laboratory, School of Biological Big Data, Yunnan Agricultural University, Kunming, China; ^5^Shenzhen Qianhai Shekou Free Trade Zone Hospital, Shenzhen, China; ^6^State Key Laboratory of Non-Food Biomass and Enzyme Technology, Guangxi Academy of Sciences, Nanning, Guangxi, China; ^7^State Key Laboratory for Conservation and Utilization of Bio-Resources in Yunnan, Yunnan Agricultural University, Kunming, China; ^8^Yunnan Research Institute for Local Plateau Agriculture and Industry, Kunming, China

**Keywords:** *Camellia sinensis*, SNPs, whole genome re-sequencing, origin, population structure

## Abstract

Phylogeographic, population genetics and diversity analysis are crucial for local tea resource conservation and breeding programs. Lincang in Yunnan has been known as the possible place of domestication for tea worldwide, yet, its genetic makeup and unique Lincang origin are little understood. Here, we reported a large-scale whole-genome resequencing based population genomic analysis in eight main tea-producing areas of Lincang in Yunnan (1,350 accessions), and the first comprehensive map of tea genome variation in Lincang was constructed. Based on the population structure, tea sample in Lincang was divided into three subgroups, and inferred Xigui and Nahan Tea Mountain in Linxiang, Baiying Mountain Ancient Tea Garden in Yun, and Jinxiu Village of Xiaowan Town in Fengqing, which belong to the birthplace of the three subgroups, were all likely to be the origin center of Lincang tea. Meanwhile, the history population sizes analysis show that similar evolutionary patterns were observed for the three subgroups of Lincang. It also was observed that the hybrid among eight areas of Lincang was noticeable, resulting in insignificant genetic differentiation between geographical populations and low genetic diversity. The findings of this study clarified the genetic make-up and evolutionary traits of the local population of tea, which gave some insight into the development of Lincang tea.

## Introduction

The tea plant (2*n* = 30; *Camellia sinensis*), a member of the genus *Camellia* (*Theaceae*), is one of the world’s important economic crops (Mondal et al., 2004). It is believed that the origin of the tea plant could trace back to southwestern China, including Yunnan Province and the adjacent areas, from which the tea spread around the world mainly through the seas and lands of the Silk Road (Kingdom-Ward, 1950; Hasimoto, 2001; Chen et al., 2005; Willson and Clifford, 2012). In the southwest China, tea plants are mainly distributed in the middle and lower reaches of the Lancang River basin, which is the upper reaches of the Mekong River. The diversity of ecological characteristics has given birth to the rich resources of ancient tea. Particularly, Lincang belongs to the second-largest tea-producing region in the Yunnan province, with the world’s oldest tea tree growing for 3,200 years, of which the Bingdao Tea Mountain, Daxue Mountain, Baiying Mountain, Nahan, and Xigui Tea Mountain are abundant for ancient tea tree resources (Ochanda et al., 2015).

In the study of the genetic evolution of tea trees, the “two-origin theory” of tea tree is the most common (Zhang et al., 2018), researchers infer that the tea originated from the two major classifications of tea, *C. sinensis* var. *assamica* (CSA) in temperate regions and *C. sinensis* var. *sinensis* (CSS) in tropical and subtropical regions. The large-leaf tea tree (CSA) originated in southwestern China or the Assam region of India, whereas the small-leaf tea originated in the south-eastern region of China (Zhang et al., 2018). For CSA, it is noted to have appeared in subtropical areas, such as eastern Yunnan, during the Miocene period of the Tertiary. The spread to the northeast in the direction of low relief began at the time of the collision of the Indian and Asian plates, after which the present crescent-shaped distribution of wild tea in Sichuan and Chongqing was formed (Kan, 2013). However, as the main domestication center of the tea plant in Lincang of Yunnan-Guizhou Plateau, where the world’s oldest tea trees are located, a great deal of uncertainty remains about the genetic structure of tea among the different tea-producing regions. Therefore, the study of genetic variation among different tea-producing regions in Lincang is essential for tea plant diversity.

To get a comprehension of the genetic structure and distribution of tea in Lincang, the single nucleotide polymorphic (SNP) markers were applied, which was identified through whole-genome resequencing (WGR) at the population level. Currently, WGR has been widely used in rice (Huang et al., 2012), maize (Hufford et al., 2012), grapes (Liang et al., 2019), and apples (Duan et al., 2017), and other important crops and economic crops. Based on WGR, Ren et al. (2021) studied 110 cannabis (*Cannabis sativa*) germplasm resources from around the world and identified candidate genes associated with differentiation traits during the domestication of hemp-type and drug-type cultivars of cannabis, revealing the domestication origin and evolutionary history of cannabis. In addition, Zhao et al. (2021) analyzed 427 Moso bamboo from 15 representative geographical areas, and constructed a genomic variation map of Moso bamboo for population evolutionary analysis, revealing the population diversity of this asexually reproducing species, etc. The previous study made it possible to identify that resequencing analysis can be used as a method to explore the origin and population structure of the tea plant in Lincang. Meanwhile, the genomes of two major variants of tea, *viz.*, big-leaf tea [Yun Kang No.10 (Xia et al., 2017)] and small-leaf tea [Shuchazao (Wei et al., 2018; Xia et al., 2020); Biyun (Zhang et al., 2020a); Longjing 43 (Wang et al., 2020); and wild tea (DASZ (Zhang et al., 2020b)], have been sequenced till now, providing a solid foundation for the large-scale application of WGR.

Herein, we collected and sequenced samples of 1,235 tea accessions from Lincang in the Yunnan province and 115 tea accessions from other regions in China. With the identified single-nucleotide polymorphisms (SNPs), we divided the tea samples from eight tea regions in Lincang into three subgroups based on the population structure, and inferred three possible origins in Lincang. In addition, It also was observed that the hybrid among eight areas of Lincang was noticeable, resulting in insignificant genetic differentiation between geographical populations and low genetic diversity. A large number of variations identified not only provide deeper insights into the genetic evolution and structural characteristics of the local tea populations but also lay a foundation for conservation and breeding programs of tea resources in Lincang, Yunnan province, China.

## Materials and methods

### Sample collection

**Figure 1:**
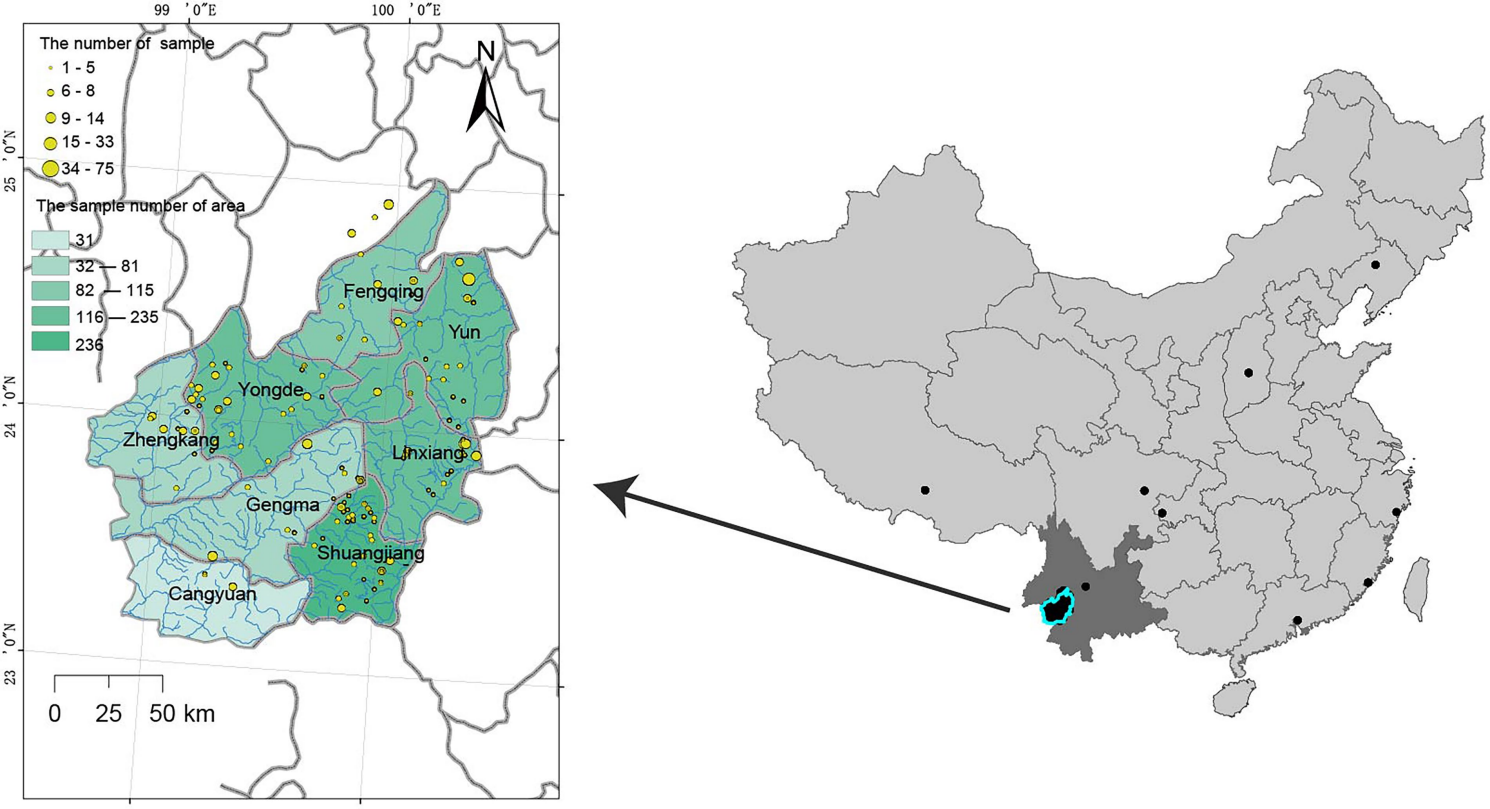
The geographical distribution of tea accessions was assessed in the present study.

A total of 1,350 tea accessions were collected at diverse sites from Lincang, Yunnan, and other regions of China during the period from 2019 to 2020. Among them, 31 samples were collected in Cangyuan, 115 in Fengqing, 78 in Gengma, 235 in Linxiang, 291 in Shuangjiang, 187 in Yongde, 217 in Yun, 81 in Zhenkang, and 115 in Others. The Others group was used as a control for the samples of the eight geographic populations, so the sampling range of the Others group was widely dispersed (Figure 1: black dot). Further, the accession KM6 (*Camellia Cuspidata*) was collected to use as an outgroup during phylogenetic analysis. The details about the sampled populations are presented in Supplementary Table 1 and the geographical distribution of these points is depicted in Figure 1.

### DNA isolation, sequencing and processing of raw read

The total DNA was extracted using the DNA secure plant kit (TIANGEN, Beijing), following the manufacturer’s protocol. Around 2 μg of the extracted DNA was used to construct the sequencing library for each accession using the NEBNext Ultra DNA Library Prep Kit (NEB Inc., America), following the manufacturer’s instructions. Paired-end sequencing libraries with an insert size of approximately 400 bp were sequenced on the Illumina NovaSeq 6000 platform. We used fastp v0.12.2 (Chen et al., 2018) for the removal of adaptor contamination, poly-N, and low-quality reads (reads having >40% bases and Phred score ≤20). Further, the paired-end reads with sequence lengths below 70 bp were filtered out. Thus, only high-quality cleaned reads were retained for downstream analysis.

### Variant calling and annotation

Paired-end reads were mapped to the reference genome of *Camellia sinensis* (Shuchazao; Wei et al., 2018) through BWA v0.7.17-r 1,188 (Li and Durbin, 2009) using default parameters. Conversion of SAM to BAM and exclusion of unmapped and multi-mapped reads were performed through SAMtools v1.3.1 (Li et al., 2009). Further, the duplicated reads were filtered out using Picard v2.1.1.^1^

After BWA alignment, the reads around indels were realigned. Realignment was performed with GATK 3.3-0-g37228af^2^ (McKenna et al., 2010) in two steps. First, we used the RealignerTargetCreator package to identify regions where realignment was needed, followed by a realignment of reads to these regions using IndelRealigner, and created a realigned BAM file for each accession. Then we detected the variation of each sample and obtained the original variation set file (gVCF format) through GATK Haplotype Caller, and gVCF files were further integrated to obtain population variation data.

The SNP filter expression parameters were set as: QD<2.0 || MQ<40.0 || FS>60.0 || SOR>5.0 || MQRankSum < −12.5 || ReadPosRankSum < −8.0 || QUAL <30. The InDel filter expression parameters were set as: QD<2.0 || ReadPosRankSum < −20.0 || InbreedingCoeff < −0.8 || FS>200.0 || SOR>10.0 || QUAL<30 (DePristo et al., 2011). Only insertions and deletions shorter than or equal to 40 bp were considered. Indels and SNPs with none bi-allelic, >50% missing calls and MAF < 0.005 were removed, which yielded the basic set. SNPs with MAF < 0.05, none bi-allelic, >50% missing calls were further removed for phylogenetic tree structure, genetic diversity analysis, LD decay, PCA and population structure analyses (the core set). The annotation of SNPs and InDels was performed through ANNOVAR v2015-12-14 (Wang et al., 2010) using tea genome as a reference.

### Phylogenetic analysis

The populations were clustered to assess the pattern of variation among the sampled populations. We used the whole-genome SNPs to construct the maximum likelihood (ML) phylogenetic tree with 100 bootstraps using SNPhylo v20140701 (Lee et al., 2014). *Camellia cuspidate* was used as an outgroup. Color coding of the phylogenetic tree was done through the iTOL web server.^3^

### LD, population structure, and PCA

The SNPs in LD were filtered out using PLINK v1.90b3.38 (Purcell et al., 2007) with a window of size 50 SNPs (advancing 5 SNPs at a time) and an *r*^2^ threshold of 0.5 to determine a pruned SNP set to be used in the population structure analysis. LD-based pruning reduces the effects of ascertainment bias in a relatively efficient manner (Malomane et al., 2018). Principal component analysis (PCA) was performed with the Genome-wide Complex Trait Analysis (GCTA) v1.25.3 (Yang et al., 2011), and the first three eigenvectors were plotted. LD was calculated using PopLDdecay v3.41 (Zhang et al., 2019). The pairwise *r*^2^ values within and between different chromosomes were calculated. The LD for each group was calculated using SNP pairs only from the corresponding group.

The population structure was analyzed using the ADMIXTURE v1.3 (Alexander et al., 2009) program with a block-relaxation algorithm. To explore the convergence of individuals, we predefined the number of genetic clusters K, from 2 to 9 and ran the cross-validation (CV) error procedure. Default methods and settings were used in the analyses.

### Genetic diversity analysis and population differentiation

The primary genetic diversity parameters like observed heterozygosity (*H_O_*), expected homozygosity (*H_E_*), inbreeding coefficient (*F*), the average pairwise diversity within a population (*θ*π), and Tajima’s *D* were calculated using the vcftools v0.1.13 (Danecek et al., 2011) with 100 kb sliding windows. In addition, overall genetic differentiation across populations measured by Weir and Cockerham’s estimator of *F_ST_* (Weir and Hill, 2002) was also calculated using the same software.

### Isolation by distance and environment

To assess the potential correlation of environmental and geographic variation with the tea genetic structure, the use of variables was necessary to adequately capture the general environmental and geographical differences among the Lincang regions sampled. For this purpose, we calculated the pairwise genetic distance matrix using the PLINK (v1.90b3.38). Meanwhile, we downloaded 4 environmental variables and two geographic variables available at each ecotype site (resolution 30 arc seconds) through WorldClim^4^ (Hijmans et al., 2005).

Among the four environmental variables, we selected two temperature and two precipitation variables. The temperature variables (in °C*10, expressed to the nearest tenths) included the mean diurnal range (MDR) and the mean temperature of the wettest quarter (MTW). The precipitation variables (expressed to the nearest mm) included annual precipitation (AP) and precipitation of the wettest month (PWM; Supplementary Table 10). In addition, we derived 10 additional variables from the squares (4) and cross-products (6) of the original four environment variables to explore whether non-linear environmental effects may also affect the genetic structure.

The geographic variables included LONG and LAT. These variables tended to show lower correlations with each other. We intended to reduce the redundancy inherent among highly associated variables (Hall and Beissinger, 2014). For the geographical distance matrix, we calculated the great-circle distance (the closest distance between two points on the Earth’s surface) in miles from untransformed LONG and LAT values for each pair of accessions using the “geosphere” package in R.^5^ For the environment distance matrix, we calculated an environmental distance matrix from Euclidean distances between pairs of accessions using all 14 environmental variables.

To sort out the potential effects of environmental variables and geographical isolation on genetic variation in tea tree samples, the Mantel test (Diniz-Filho et al., 2013) of correlation among genetic, environmental and geographical distance matrices was applied by using the VEGAN^6^ package in R, in which significance testing of the correlations was performed with 10,000 permutations. Then, we explored whether there was a significant association between environmental distance and genetic distance by using a partial Mantel test, adjusted for any effects of geographical distances. Significance in this test was interpreted as meaning that genetic variation among the tea was influenced by environmental selection, whereas a nonsignificant result suggested a role for isolation by distance (genetic drift).

### Differentiation and historical relationships between populations

The historical relationship between Lincang tea geographical populations was estimated using TreeMix (Pickrell and Pritchard, 2012), which uses a Maximum Likelihood (ML) method based on a Gaussian model of allele frequency change. The topology of the ML trees changes depending on the number of migration events (*m*) allowed in the model. Here we use *m* = 1 to *m* = 5 (Fitak, 2021). The bootstrap values on the tree are based on 1,000 replicates. Arrows on the graph represent admixture events between different tea populations. The Other tea population was used for roots.

### Demographic history reconstruction

To uncover the evolution history of lincang tea subpopulations, we use MSMC2^7^ to infer population size of each group. The input files for MSMC2 were generated according to MSMC Tools.^8^ In brief, only sites with uniquely mapped reads and sites with coverage depths between 0.5-fold and 2-fold of mean depth were used in the analyses. The remaining genomic regions were masked using the script bamCaller.py. Then all segregating sites within each group were phased using SHAPEIT (Version: v2.r904; Delaneau et al., 2011). A mutation rate of 6.1 × 10^–9^ per site per year was used.

### Genome scanning for selective sweep signals

RAiSD (Raised Accuracy in Sweep Detection, Version 2.9; Alachiotis and Pavlidis, 2018) was used to detect signatures of selective sweeps based on the μ statistics. The significant threshold for μ statistic score was set as top 0.1%. Then, We performed a genetic differentiation (*F_ST_*) and nucleotide polymorphism (*θ*π) based cross approach to investigate the selection signals across the whole genome. A 50 kb sliding window with 10 kb step approach was applied to quantify *F_ST_* and *θ*π by using VCFtools software (v0.1.13). The annotated genes living in these regions were considered candidate selected genes.

## Results

### Whole-genome resequencing and variant calling

Resequencing of 1,350 tea samples yielded around 9.67 Tb data (64,462,516,344 paired-end raw reads). Mapping of these reads with the reference genome resulted in an average alignment rate of 98.9 ± 2.65% (63.79–99.54%, Supplementary Figure 1 and Supplementary Table 1). The information about 9 populations with reads is presented in Table 1. The average alignment rate of the population ranged from 96.75 to 98.75%; the average number of reads per sample of the population ranged from 45,974,894 to 56,685,556;

**Table 1:** Sequencing and genetic variation information of tea from different geographic regions.

Population name	Number of samples	Total number of reads	Average number of reads per sample	Average alignment rate	Average number of SNP per sample	SNP number before basic filtering criteria	Average number of indel per sample	Indel number before basic filtering criteria
Cangyuan	31	1,757,252,234	56,685,555.94	98.46%	5,363,031.48	166,253,976	196,162.10	6,081,025
Zhenkang	81	3,723,966,416	45,974,894.02	98.67%	4,470,043.80	362,073,548	153,944.21	12,469,481
Linxiang	235	11,292,071,182	48,051,366.73	98.38%	4,460,338.49	1,048,179,546	157,390.56	36,986,781
Yun	217	10,644,281,662	49,051,989.23	98.19%	4,424,562.90	960,130,150	150,987.73	32,764,338
Gengma	78	4,006,235,570	51,361,994.49	97.77%	4,972,003.38	387,816,264	182,226.69	14,213,682
Fengqing	115	5,566,575,542	48,405,004.71	98.21%	4,424,233.28	508,786,827	151,856.77	17,463,528
Yongde	187	8,770,015,522	46,898,478.73	96.75%	4,386,209.56	820,221,187	156,149.85	29,200,022
Shuangjiang	291	13,270,042,504	45,601,520.63	98.75%	4,499,634.64	1,309,393,681	158,839.54	46,222,305
Other	116	5,432,075,712	45,229,542.50	97.55%	3,488,540.00	404,670,652	111,968.00	12,988,316

After applying basic filtering criteria (see “Materials and Methods” section), we identified 356,171,898 SNPs and 27,367,688 short genomic insertions and deletions (indels). The result of filtering is presented in Supplementary Figures 2, 3 and Supplementary Tables 2, 3. Further filtering yielded a core set of 27,550,879 SNPs and 1,139,750 indels (≤40 bp) with minor allele frequency (MAF) more than 0.05 and max missing less than 0.5. The information about SNPs and InDels is presented in Table 1 and Supplementary Table 4. Meanwhile, in the core data set, 91.36% (25,170,861) of the SNPs were located in the intergenic region, 4.82% (1,327,890) in the intronic region, 1.31% (36,049) in the 5′-UTR, and 2.95% (81,272) in the 3′-UTR. We observed 0.91% (251,002) and 1.01% (277,265) of the SNPs in the upstream and downstream regions of the genes, respectively. Further, 0.0151% (4,150) were located in the variable splicing region and 1.44% (395,863) were present in the exonic regions of the genes (Table 2 and Supplementary Figure 4). Among the exonic SNPs, the proportion of non-synonymous SNPs was found to be 56.31% (223,048) and that of synonymous SNPs was found to be 41.60% (164,772), with the non-synonymous to synonymous mutation ratio of 1.353. The total number of stop-gain SNP mutations was 7,652, whereas the total number of stop-loss SNP mutations was 557 (0.00202%). In addition, 950,567 indels were found to be located in the intergenic region (83.40%), followed by the intronic region (Intronic) with 118,677 (10.41%; Table 3 and Supplementary Figure 4).

**Table 2:** The number of SNPs and indels in different genome structures.

Variants	Type	Core set
SNP	Total	27,550,879
	Intergenic	25,170,861
	Intronic	1,327,890
	Exonic	395,863
	5′-UTR	36,049
	3′-UTR	81,272
	UTR5; UTR3	237
	Upstream	251,002
	Downstream	277,265
	Upstream; downstream	6,095
	Splicing	4,150
	Exonic; splicing	195
Indel	Total	1,139,750
	Intergenic	950,567
	Intronic	118,677
	Exonic	14,064
	5′-UTR	3,715
	3′-UTR	8,271
	UTR5; UTR3	9
	Upstream	19,862
	Downstream	23,608
	Upstream; downstream	569
	Splicing	399

**Table 3:** The number of large-effect SNPs and indels.

Variants	Type	Core set
SNP	Total (exonic+exonic splicing)	396,058
	Nonsynonymous	223,048
	Synonymous	164,772
	Nonsyn/syn ratio	1.354
	Stop-gain	7,652
	Stop-loss	557
	Unknown	29
Indel	Total (exonic+exonic splicing)	14,073
	Frameshift deletion	6,189
	Frameshift insetion	3,744
	Non-frameshift deletion	2,573
	Non-frameshift insertion	1,300
	Stop-gain	245
	Stop-loss	22

### Population structure and principal component analysis (PCA)

**Figure 2:**
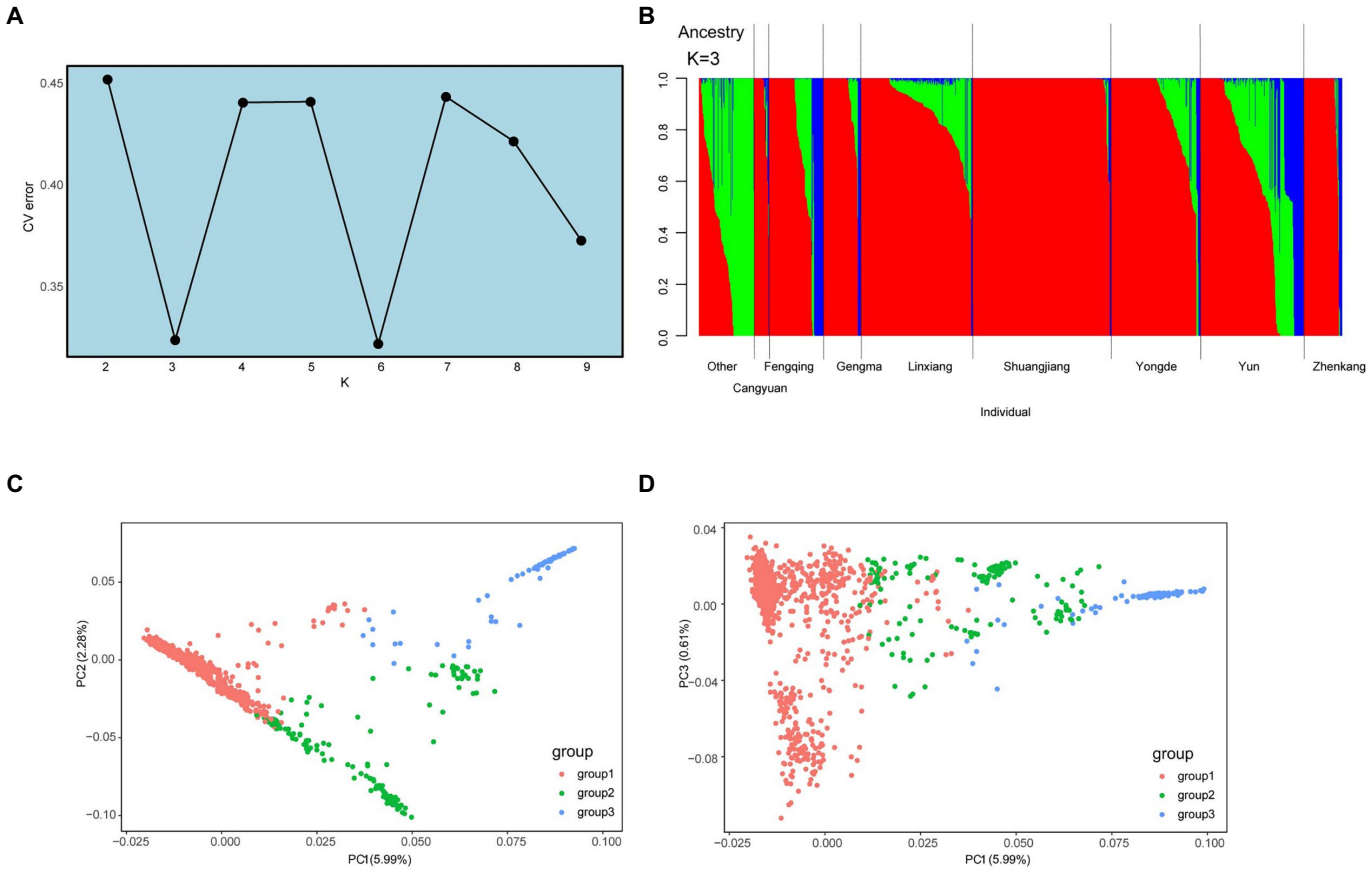
**(A)** Cross-validation errors. The x-axis represents the *K* value, while y-axis indicates the cross-validation errors. The dot shows *K* = 3 with the lowest cross-validation errors. **(B)** Population structure of the tea plant collections, which represents the best inferred K-value with the lowest cross-validation errors. **(C,D)** PCA analysis. From left to right, the three squares indicate group I, group II, and group III.

The SNPs after filtering linkage disequilibrium sites (MAF > 0.05) were used to analyze the population structure and differentiation. According to the calculated CV error value, when *K* = 3, the CV error value is the smallest (Figure 2A). Therefore, there are three genetic stocks (genetically different populations) represented as red, green, and blue color (Figure 2B) in Lincang. Interestingly, Shuangjiang and Zhenkang populations were found to be almost genetically pure, whereas the other populations showed a substantial level of genetic admixtures (Figure 2B). The red genetic stock appears to be the most dominant followed by green, whereas the third genetic stock, blue, represents a very small proportion only in the Yun, Zhenkang, and Fengqing tea populations.

In the principal component analysis of tea accessions from Lincang (Figure 2), the three principal components of PC1, PC2, and PC3 represented 5.99, 2.28, and 0.61% of the total genetic variance, respectively. The accessions were grouped into three subgroups based on the STRUCTURE inferred clustering result with 1,105, 172 and 74 accessions came to gather for sub-populations 1, 2, and 3, respectively (When an individual has the highest proportion of red ancestors, it is classified as group 1; then, accessions with the highest proportion of yellow ancestors were assigned into the group 2; otherwise, group 3; Figure 2B). Among them, the first PC (PC1) distantly clustered varieties from groups 2 and the combination of PC1 (5.99%) and PC2 (2.28%) can distinguish groups 2 (red, Figure 2C) from other accessions.

### Phylogenetic analysis

**Figure 3:**
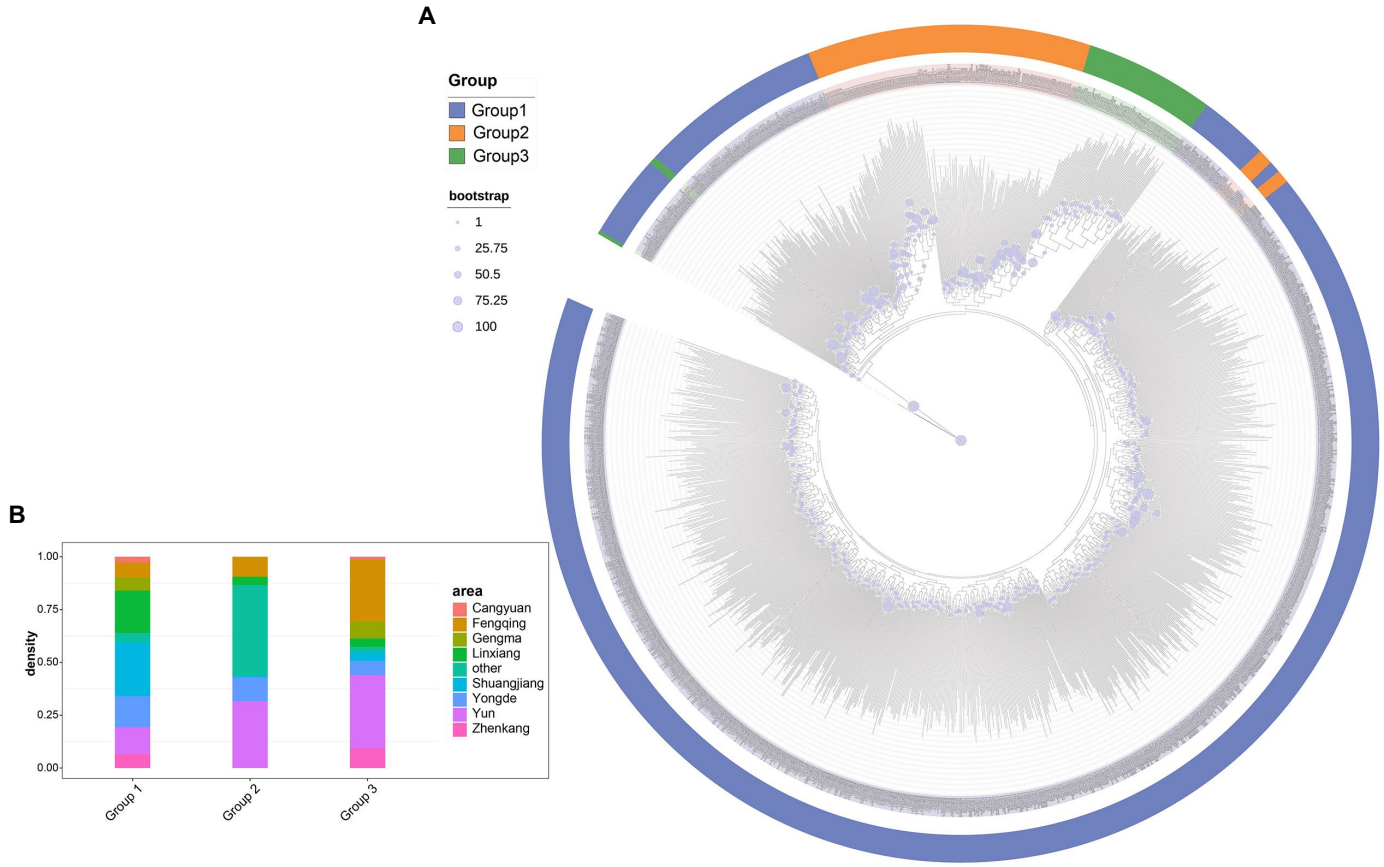
**(A)** Phylogenetic relationships of 1,350 ancient tea plants in three subgroups. Deep blue, orange, and green represent group 1, group 2, and group 3. Bootstrap values are indicated by blue circles. KM6 (*C. Cuspidata*) was selected as the outgroup. **(B)** The region distribution of accession from different tea plant subpopulations.

Based on the core SNP set, the phylogenetic tree was constructed using the maximum likelihood (ML) method with KM6 (*C. cuspidata*) as the outgroup, and the bootstrap value of 100. The result of regional phylogenetic analysis showed that no precise geographic or regional clustering was observed in the phylogenetic tree (Supplementary Figure 5). However, when tea tree samples were analyzed into three subgroups based on the results of genetic stratification analysis (Figure 3A), the phylogenetic tree showed four distinct clustering situations, which recapitulates the same patterns in the principal component analysis (PCA) and model-based clustering (Figure 3B). Among them, group 1 was divided into two clusters, and group 2 and 3 were divided into a cluster. In group 1 (Figure 3A; blue), the samples collected in Cluster 1 were dominated by Yun and Linxiang, which had a close genetic relationship with the outgroup. It can be inferred that the border between Yun and Linxiang may be the origin of tea. The samples in Cluster 2 are dominated by Shuangjiang and Linxiang, which are genetically more distant from the outgroup, and it can be inferred that the area covered by Cluster 2 may have been introduced later and thus developed into numerous new branches. In group 2 (Figures 3A,B), tea samples from Yun County were dominant, and the tea samples collected in Yun (leaf nodes) were genetically closer to the proximal ancestors (the inner node). It can be further inferred that the region of Yun is the area where the initial origin has produced differentiation afterward. In group 3 (Figure 3A: green and Figure 3B), tea samples of Fengqing were dominated and were more distantly related to the ancestor (inner node), indicating that group 3 represented by Fengqing was more divergent compared to group 1 and group 2. Notably, we found that 15 tea accessions, collected from Fengqing, Other, and Yun of group 2, were clustered with the Cluster 2 of groups 1; Moreover, 4 tea accessions from Yun in subcluster 3 were clustered into Cluster 1 of groups 1. these samples may exist with the possibility of introgression in their clustered regions.

### Genetic diversity and population differentiation

**Figure 4:**
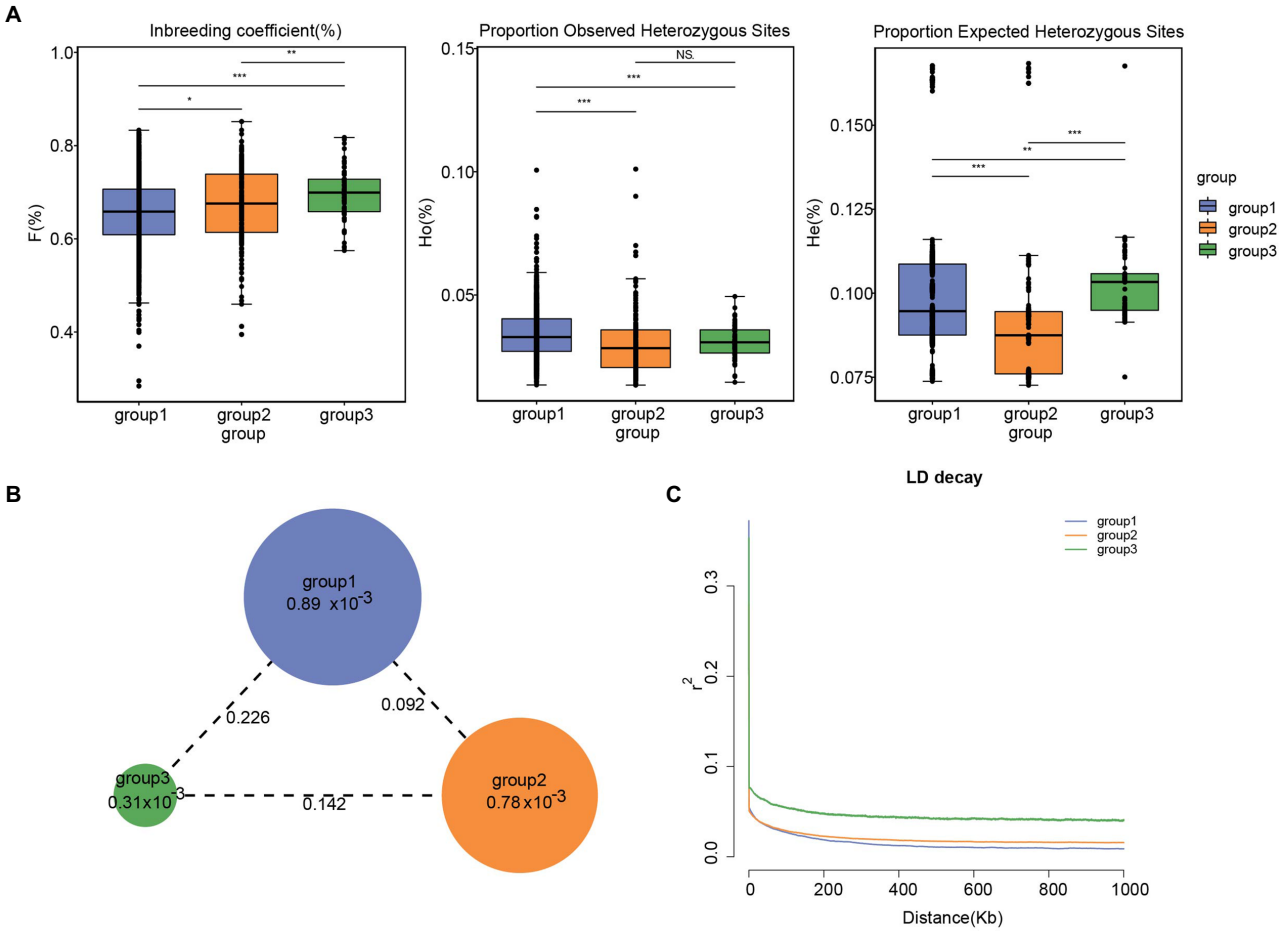
**(A)** Inbreeding coefficient, Proportion Observed Heterozygous Sites(%) and Proportion Expected Heterozygous Sites (%) estimation in tea plant subpopulations. Significances of difference between groups were derived with one-sided t-test. Among them, 0.01 < *p < 0.05; 0.001 < **p < 0.01; ***p < 0.001. **(B)** Nucleotide diversity (*θπ*) and genetic differentiation (*F_ST_*) within different tea plant subpopulations calculated using the sliding-window approach (100 kb windows with 100 kb steps). The circle size represents the mean value of *θπ* in each subpopulation. The numbers marked between each subpopulation indicate the mean value of *F_ST_*. **(C)** LD decay in different subpopulations. The x-coordinates indicate the distance between bases and the y-coordinates indicate the mean value of the correlation coefficient.

The parameters *θπ*, *H_O_*, *H_E_*, *F*, and Tajima’s *D* were calculated for tea accessions to estimate the patterns of genetic diversity. The primary genetic diversity parameters are presented in Supplementary Tables 5–9, Table 4 and Figure 4. For the subgroup, the expected heterozygosity (*H_E_*) of the tea populations varied between 9.04 (group 2) and 10.25% (group 3), while the observed heterozygosity (*H_O_*) of the tea populations ranged between 3.05% (group 2) and 3.43% (group 3). The inbreeding coefficient (*F*) of the tea populations varied between 65.52% (group 1) and 69.66% (group 3). It is worth noting that a degree of variability existed between subgroups in both *H_E_* and *F* (Figure 4A). Nucleotide diversities (*θπ*) in the three subgroup were estimated at the individual level after the correction for sample size. The analysis found that group 1 (8.89 × 10^−4^) had the highest π value, while group 3 (3.31 × 10^−4^) had the lowest π value (Table 4 and Figure 4C). Meanwhile, tajamaD analysis showed positive Tajima’s D test values for all subgroup in Lincang (group 3 < group 2 < group 1), in agreement with the findings of π analysis, revealed that the Lincang tea populations may be experiencing group constriction, which may be related to the directional selection. For the regional population(Supplementary Figure 6; Supplementary Table 9), Cangyuan population had the highest nucleotide diversity (1.777 × 10^−3^), which is consistent with the expected heterozygosity and the observed heterozygosity. Otherwise, Tajama’D of Cangyuan is closest to 0 concerning the other eight populations, indicating that Cangyuan is the least selected in Lincang and preserves a large amount of tea germplasm, which is potential resources to expand the genetic resources of improvement. The lowest level of diversity was found for the Shuangjiang population (1.071 × 10^−3^), which resulted from a long history of breed formation and selective breeding more than in most other areas.

**Table 4:** Genetic diversity of subgroups of lincang tea.

Group	*F*(%)	*H*_E_	*H*_O_	*θπ*	Tajama *D*
Group 1	65.52%	9.87%	3.43%	8.89E-04	2.41
Group 2	67.21%	9.04%	3.05%	7.80E-04	1.41
Group 3	69.66%	10.25%	3.12%	3.13E-04	0.36

The pairwise *F_ST_* between individual populations is presented in Figure 3C. Our analysis revealed that group 1 and 2 (0.092) are more closely distant genetically, while group 1 and 3 (0.226) are more distant from each other. It is inferred that the ancestral population of subpopulation 3 may have diverged prior to the ancestral populations of subpopulations 1 and 2, resulting in greater genetic divergence. ln addition, *F_ST_* values between regions indicate a weak genetic differentiation between regional populations (Supplementary Figure 7).

### Isolation by distance and environment

In the Mantel tests, we can reject the null hypothesis that these three matrices, genetic distance and environment distance (*r*_1_ = 0.1462), genetic distance, and geographic distance (*r*_2_ = 0.07843), are moderately related with alpha = 0.01 (all *p* ≤ 0.0001). The observed correlation (*r*_1_ = 0.1462, *r*_2_ = 0.07843) suggests that the matrix entries are positively associated. This means that the larger geographical distances between tea accessions lead to greater genetic differences, and the higher differences in environmental variables result in more genetic distance.

In the partial Mantel test, the genetic and environmental distances still were moderate correlated (*r*_3_ = 0.09541, *p* = 0.001) after considering geographic distance. This result suggests that environmental selection has a weak but non-negligible effect in shaping genetic variation in wild tea germplasm after individual control distance isolation. It can be inferred that there may be introductions of the tea accessions from each other resulting in the weak regional characteristics of tea trees in these populations, which is corresponding to the results of regional phylogenetic analysis (Supplementary Figure 5).

### Linkage disequilibrium analysis

Linkage (LD) is a non-random combination of alleles at different positions in a given population, and usually expressed as *D* and *r*_2_ values (Slatkin, 2008), which are mainly related to whether the same species have experienced domestication pressure, regional selection pressure, and nucleotide diversity. The LD analysis showed that the *r*_2_ values of accessions of all subgroups are <0.4 (Figure 4C). When the *r*_2_ value is <0.4, it is generally considered that there is no effective linkage or no linkage. At the same time, Linkage disequilibrium decay distance indicated that the degree of Linkage in Lincang is pretty low, which is consistent with the information that the tea samples is basically ancient tea trees with a short history of artificial cultivation.

### Gene flow and historical effective population size

**Figure 5:**
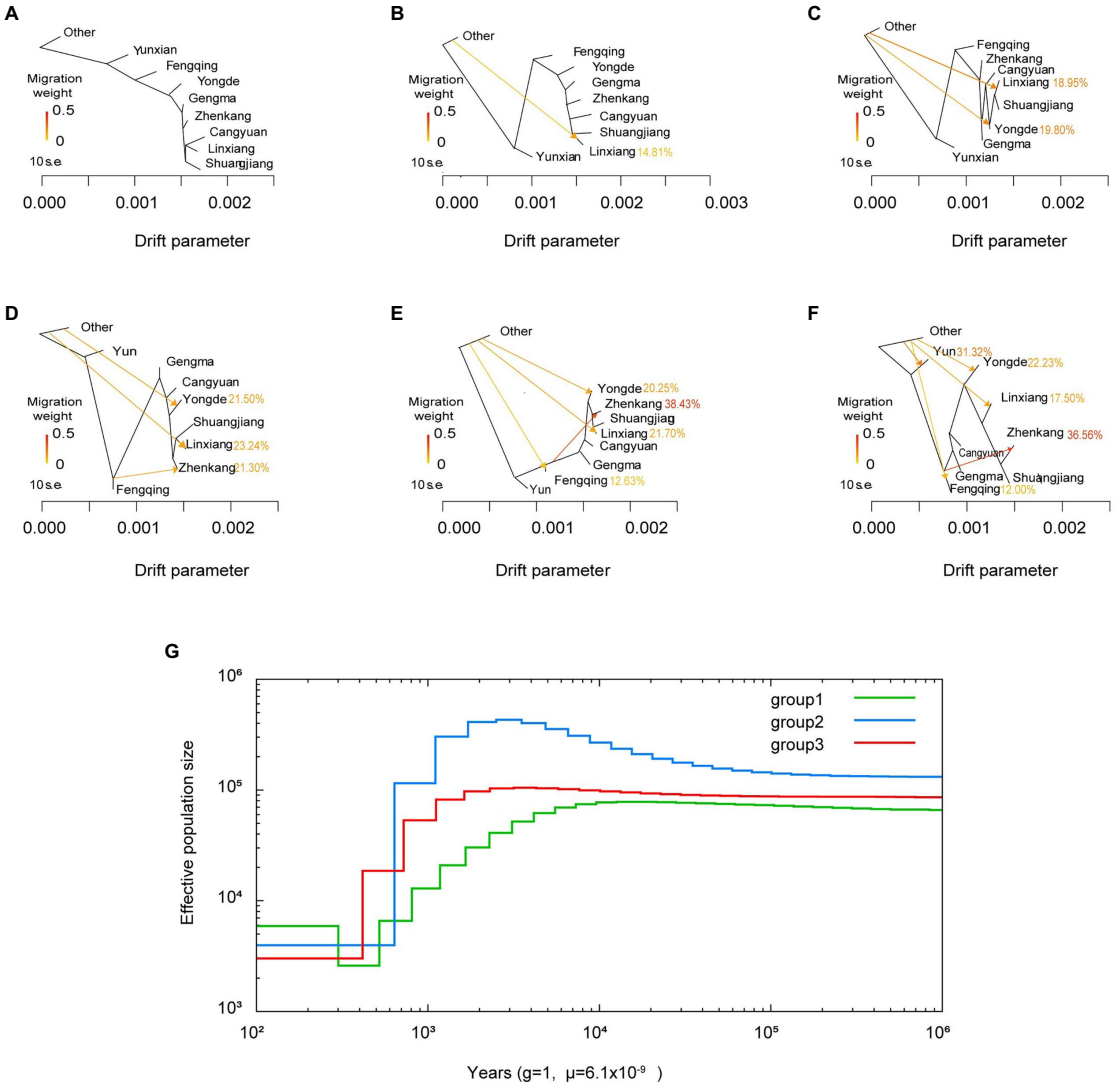
**(A–F)** Population splits and migrations between tea plant accessions. Hybridization likely occurs among the different areas in lincang. **(G)** MSMC-derived demographic history of different tea plant subpopulations from 10^2^ to 10^6^  years ago.

Population history includes events such as population bottlenecks, expansions, migrations and admixtures, which have important implications for the formation of genetic polymorphic patterns in populations. To reveal the evolutionary history of Lincang tea, we applied the multiple sequentially Markovian coalescent (MSMC; Schiffels and Durbin, 2014) model to the analysis of phased SNP data from three subgroups. As displayed in Figure 5H, similar evolutionary patterns were observed for the three subgroups of Lincang. Group 1 (green line) and group3 (red line) manifested a slight Ne expansion around 10–1,000 Kya, 2–100 Kya and a subsequent Ne contraction (Ne ≈ 80,000 down to Ne ≈ 1,800 and Ne ≈ 100,000 down to Ne ≈ 2,000) around 0.5–10 Kya, 0.4–3 Kya, respectively. Interestingly, the range of expansion and contraction in group 2 is significantly greater compared to groups 1 and 2, inferring that group 3 is likely to be more heavily influenced by human and environmental influences.

Also, TreeMix was used for inferring historical segregation and admixture of populations based on genome-wide SNP allele frequency data. Regarding the changes in tea gene flow, Figures 5E,F both show that there is a Treemix vector connected Fengqing to Zhenkang, and the proportion of gene exchange is relatively large, 38.43 and 36.57%, respectively, indicating bidirectional gene flow. This indicates that hybridization likely occurs among the different geographic areas in nature.

### Selection signals of Lincang tea subgroups

**Figure 6:**
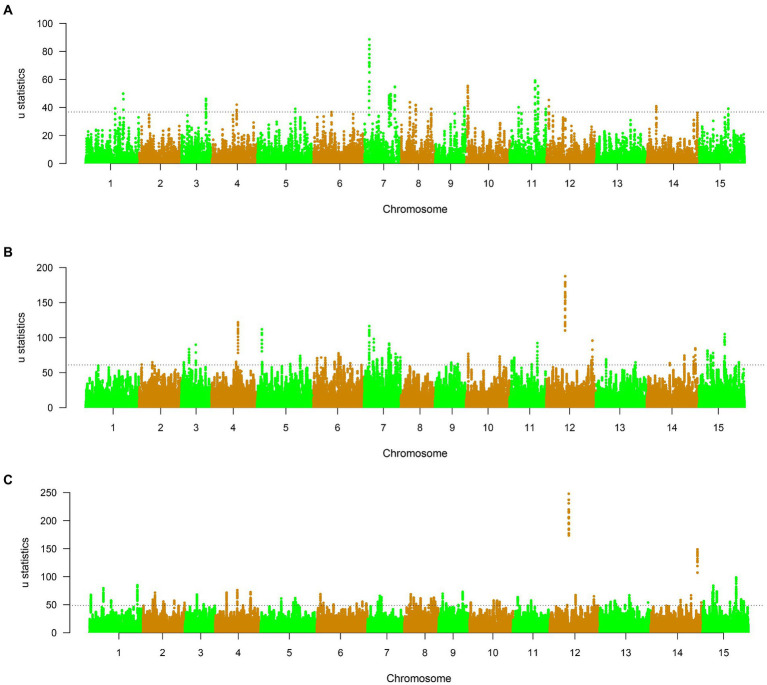
**(A–C)** μ statistics calculated by RAiSD across the genome in different tea plant subpopulations. The dashed lines mark the regions at the top 0.1%.

To study the selective characterization of three subgroups of Lincang, 611, 1,328, and 2,090 selective sweep regions were both detected in different tea plant subpopulations, which harbored 65,145 and 201 candidate genes, respectively (Supplementary Tables 10–13, Figure 6, and Supplementary Figures 9–11). In group 1, the highest signal 88.62 was found on chromosome 7 at position 26,812,875 bp, and the most selective signals were detected on chromosome 7 (278 signals). In group 2, 344 selective signals were identified on chromosome 6, and the highest signal reached to 187.6 on chromosome 12 at 96,426,312 bp. For group 3, chromosome 15 (354 selective regions) has the most selective regions, and chromosome 12 at 96,038,055 bp has the most selective signal 247.9. GO enrichment of the 65,145 and 201 domesticated candidate genes of three subgroups showed significant functional representation in the GO categories of negative regulation of cellular process in group 1; peptidase S8/S53 domains, oxidoreductase activity, Golgi apparatus, obsolete oxidation–reduction process, cellular response to lipid in group2; and UDP-glucosyltransferase activity and glucosyltransferase activity in group3 (Supplymentary Figure 12). Interestingly, The peptidase S8/ S53 domains enriched in subpopulation 2 are functionally consistent with those enriched in previous studies of small-leaf tea (Wang et al., 2020), and it is inferred that subpopulation 2 may contain domesticated and hybrid species of small-leaf tea. On the drawback, the lack of functional clarity of many high-signal positional candidate genes makes it impossible to identify selective differential traits between different subpopulations.

## Discussion

In this study, we reported the characteristics of whole-genome SNPs and indels of 1,350 tea accessions, covering almost all varieties of tea in Lincang, Yunnan. The genomic variation data on the scale of this study is the largest ever reported for the tea plant. In total, our study generated 64,462,516,344 short reads and 5.967 billion SNPs, and together these datasets provide the most extensive genomic resource available for tea researchers.

In the analysis of population structure, genetic stratification analysis by the Bayesian clustering model revealed the presence of three subpopulations. The study found that samples from group 1 were predominantly from Yun and Linxiang, and based on the geographical distribution of the sample, it can be inferred that the origin of group 1 is most likely from the Xigui and Nahan tea mountains located at the junction of Yun and Linxiang. It also has been identified that the wild tea plantation in the Xigui and Nahan tea mountains is one of China’s tea tree origin centers (Zhou and Zhu, 2007), which is consistent with the analysis of phylogenetic trees. Further, the border of Linxiang and Gengma, located in the northern part of Shuangjiang County, maybe the main site of divergence for cluster 2 of the Lincang tea tree group 1 (Figure 3B). It is home to the north–south branch of the Hengduan Mountain System-Bangma mountain, whose main peak is the Mengku snow mountain, with the highest and largest wild ancient tea tree community in the world at an altitude of 2,200–2,750 m. The tea samples collected from Linxiang, Shuangjiang and Gengma probably originated in the wild ancient tea garden of Daxue mountain in Mengku. Meanwhile, it is proved that the Mengku tea species are mainly distributed in Linxiang, Cangyuan and Gengma by introductions centered on Shuangjiang County (Chen, 1984), which is further support for the area distribution of samples in group 1 (Figure 3B).

Likewise, Baiying Mountain’s ancient tea plantations of Manwan Town, located in the north-east of Yun, are home to a mixture of Dali tea, Assam tea (*C. sinensis* var. *assamica*), and intermediate species of tea trees (Zhao et al., 2014), known as the world’s tea gene pool. And is likely to contribute to the main origin of group 2 regional characteristics. Moreover, it also was found in the study that samples mainly from Fengqing and Yunxian, with a few from other Lincang areas, comprise group 3 (Figure 3B: blue), leading to a tentative inference that the origin of group 3 may be located in the Fengqing or Yun areas. Due to the influence of introgression among samples and the effectiveness of its genetic variation in this study, the possibility of other conditions cannot be ruled out. A more refined tea sample analysis can be carried out. Furthermore, details about development routes of different tea species subpopulations from Lincang tea region still need to be further clarified.

In addition, MSMC analysis identify a dramatic expansion and contraction of effective population size of the different tea subgroups of Lincang. It is noted that the Pu people began domesticating and using wild tea trees in Yunnan 3,000 years ago during the Shang and Zhou periods, which may account for the lower effective population. On the genetic variability of the Lincang tea, it was found the significant genetic differences in the three subgroups and the moderate level of genetic differentiation among eight regional populations. Meanwhile, genetic difference and environment (*r*_1_ = 0.1462), and geographic (*r*_2_ = 0.07843) were not significantly associated, indicating that introgressive hybridization and artificial selection may have occurred between regions, in agreement with the results of gene flow analysis and local tea plantation policies. ln addition, the apparent introduction of Lincang tea trees and close geographical distances between sampling regions have led to no clear classification between geographical regions, making the classification of Lincang tea somewhat challenging. In the coming years, further worldwide sampling and analysis will help resolve the current debates on tea taxonomy.

Moreover, a low level of diversity was observed in Lincang, contrary to previous research on tea trees in Lincang (Mao, 2018). One possible explanation of the result is that sampling led to differences in genetic diversity. Previous studies have focused on wild teas, whereas this study was based on both wild and cultivated teas, with a much wider distribution of sampling sites, which makes Lincang’s genetic diversity more convincing. Another, compared to other species, the nucleotide diversity of these populations also is lower than that of common wild rice (3 × 10^−3^; Huang et al., 2012), wild soybean (2.94 × 10^−3^; Zhou et al., 2015), wild grape (3.5 × 10^−3^; Liang et al., 2019), etc. This may be related to the relatively small effective population of the group in the Lincang tea region. Thus, the current situation of genetic diversity in Lincang should be a cause for widespread concern and protection for the local people.

In conclusion, our population genomic investigations of Lincang tea provide novel information about their ancestry, gene flow, history of effective population size, and genetic diversity. The Lincang tea has three distinct possible origins: Xigui and Nahan Tea Mountain in Linxiang, Baiying Mountain Ancient Tea Garden in Yun, and Jinxiu Village of Xiaowan Town in Fengqing. The finding of the origin location in the Lincang region offers a theoretical benchmark for the investigation of the genesis and development of tea plants on a more global scale. Furthermore, the studies into numerous facets of tea plant biology will be made easier thanks to this extensive SNP database of tea species.

## Data availability statement

The raw sequencing data reported in this paper have been deposited in the BIG Data Center (http://bigd.big.ac.cn/gsa) under the accession number PRJCA011312. In addition, the sequencing data are also accessible from the tea database (http://teabase.ynau.edu.cn/index/download/index).

## Author contributions

YD designed the study. ZW, SN, GX, LY, CW, DL, ShiZ, SY, and ShuZ collected the tea samples. SD, YL, and LY performed the genome data analyses. YL and LY wrote the manuscript. YD, LK, SD, and JS revised and improved the manuscript. All authors reviewed and approved the final version of the manuscript.

## Funding

This work was supported by Digitalization of Biological Resource Project (grant number 202002AA100007), Yunnan; Yunnan provincial key programs of Yunnan Eco-friendly Food International Cooperation Research Center project (grant number 2019ZG00908); and Yunnan Provincial Science and Technology Department Project (Development and Application of Biological Resource Digitalization; grant number 2019008).

## Conflict of interest

The authors declare that the research was conducted in the absence of any commercial or financial relationships that could be construed as a potential conflict of interest.

## Publisher’s note

All claims expressed in this article are solely those of the authors and do not necessarily represent those of their affiliated organizations, or those of the publisher, the editors and the reviewers. Any product that may be evaluated in this article, or claim that may be made by its manufacturer, is not guaranteed or endorsed by the publisher.

## Footnotes

^1^https://sourceforge.net/projects/picard/ (Accessed August 26, 2022).

^2^https://gatk.broadinstitute.org/ (Accessed August 26, 2022).

^3^http://itol.embl.de (Accessed August 26, 2022).

^4^https://www.worldclim.org/ (Accessed August 26, 2022).

^5^http://cran.r-project.org/web/packages/geosphere/index.html (Accessed August 26, 2022).

^6^http://CRAN.Rproject.orgd/package=vegan (Accessed August 26, 2022).

^7^https://github.com/stschiff/ msmc2 (Accessed August 26, 2022).

^8^https://github.com/stschiff/msmc-tools (Accessed August 26, 2022).

## References

[ref1] Alachiotis N. Pavlidis P. (2018). RAiSD detects positive selection based on multiple signatures of a selective sweep and SNP vectors. Commun. Biol. 1, 1–11. doi: 10.1038/s42003-018-0085-8 30271960 PMC6123745

[ref2] Alexander D. H. Novembre J. Lange K. (2009). Fast model-based estimation of ancestry in unrelated individuals. Genome Res. 19, 1655–1664. doi: 10.1101/gr.094052.109, PMID: 19648217 PMC2752134

[ref3] Chen C. (1984). A general history of tea industry. Agricultural Press.

[ref4] Chen J. Wang P. Xia Y. Xu M. Pei S. (2005). Genetic diversity and differentiation of Camellia sinensis L. (cultivated tea) and its wild relatives in Yunnan province of China, revealed by morphology, biochemistry and allozyme studies. Genet. Resour. Crop Evol. 52, 41–52. doi: 10.1007/s10722-005-0285-1

[ref5] Chen S. Zhou Y. Chen Y. Gu J. (2018). Fastp: an ultra-fast all-in-one FASTQ preprocessor. Bioinformatics 34, i884–i890. doi: 10.1093/bioinformatics/bty560, PMID: 30423086 PMC6129281

[ref6] Danecek P. Auton A. Abecasis G. Albers C. A. Banks E. DePristo M. A.. (2011). The variant call format and VCFtools. Bioinformatics 27, 2156–2158. doi: 10.1093/bioinformatics/btr330, PMID: 21653522 PMC3137218

[ref7] Delaneau O. Marchini J. Zagury J. F. (2011). A linear complexity phasing method for thousands of genomes. Nat. Methods 9, 179–181. doi: 10.1038/nmeth.1785, PMID: 22138821

[ref8] DePristo M. A. Banks E. Poplin R. Garimella K. V. Maguire J. R. Hartl C.. (2011). A framework for variation discovery and genotyping using next-generation DNA sequencing data. Nat. Genet. 43, 491–498. doi: 10.1038/ng.806, PMID: 21478889 PMC3083463

[ref9] Diniz-Filho J. A. F. Soares T. N. Lima J. S. Dobrovolski R. Landeiro V. L. Telles M. P. D. C.. (2013). Mantel test in population genetics. Genet. Mol. Biol. 36, 475–485. doi: 10.1590/S1415-47572013000400002, PMID: 24385847 PMC3873175

[ref10] Duan N. Bai Y. Sun H. Wang N. Ma Y. Li M.. (2017). Genome re-sequencing reveals the history of apple and supports a two-stage model for fruit enlargement. Nat. Commun. 8, 1–11. doi: 10.1038/s41467-017-00336-7 28811498 PMC5557836

[ref11] Fitak R. R. (2021). Opt M: estimating the optimal number of migration edges on population trees using Treemix. Biol. Methods Protoc. 6:bpab017. doi: 10.1093/biomethods/bpab017, PMID: 34595352 PMC8476930

[ref12] Hall L. A. Beissinger S. R. (2014). A practical toolbox for design and analysis of landscape genetics studies. Landsc. Ecol. 29, 1487–1504. doi: 10.1007/s10980-014-0082-3

[ref13] Hasimoto M. (2001). "The origin of the tea plant", in: *Proceedings of 2001 International Conference on O–Cha (Tea) Culture and Science (Session II)*, 5–8.

[ref14] Hijmans R. J. Cameron S. E. Parra J. L. Jones P. G. Jarvis A. (2005). Very high resolution interpolated climate surfaces for global land areas. Int. J. Climatol. 25, 1965–1978. doi: 10.1002/joc.1276

[ref15] Huang X. Kurata N. Wang Z.-X. Wang A. Zhao Q. Zhao Y.. (2012). A map of rice genome variation reveals the origin of cultivated rice. Nature 490, 497–501. doi: 10.1038/nature11532, PMID: 23034647 PMC7518720

[ref16] Hufford M. B. Xu X. Van Heerwaarden J. Pyhäjärvi T. Chia J.-M. Cartwright R. A.. (2012). Comparative population genomics of maize domestication and improvement. Nat. Genet. 44, 808–811. doi: 10.1038/ng.2309, PMID: 22660546 PMC5531767

[ref17] Kan N. C. (2013). Study on the Origin of Tea Tree and the Distribution of Wild Tea Tree in Sichuan and Chongqing. Southwest J. Agr. 26, 382–385. doi: 10.16213/j.cnki.scjas.2013.01.056

[ref18] Kingdom-Ward F. (1950). Does wild tea exist? Nature 165, 297–299. doi: 10.1038/165297a0

[ref19] Lee T.-H. Guo H. Wang X. Kim C. Paterson A. H. (2014). SNPhylo: a pipeline to construct a phylogenetic tree from huge SNP data. BMC Genomics 15, 1–6. doi: 10.1186/1471-2164-15-162 24571581 PMC3945939

[ref20] Li H. Durbin R. (2009). Fast and accurate short read alignment with burrows–wheeler transform. Bioinformatics 25, 1754–1760. doi: 10.1093/bioinformatics/btp324, PMID: 19451168 PMC2705234

[ref21] Li H. Handsaker B. Wysoker A. Fennell T. Ruan J. Homer N.. (2009). The sequence alignment/map format and SAMtools. Bioinformatics 25, 2078–2079. doi: 10.1093/bioinformatics/btp352, PMID: 19505943 PMC2723002

[ref22] Liang Z. Duan S. Sheng J. Zhu S. Ni X. Shao J.. (2019). Whole-genome resequencing of 472 Vitis accessions for grapevine diversity and demographic history analyses. Nat. Commun. 10, 1–12. doi: 10.1038/s41467-019-09135-8 30867414 PMC6416300

[ref23] Malomane D. K. Reimer C. Weigend S. Weigend A. Sharifi A. R. Simianer H. (2018). Efficiency of different strategies to mitigate ascertainment bias when using SNP panels in diversity studies. BMC Genomics 19, 1–16. doi: 10.1186/s12864-017-4416-9 29304727 PMC5756397

[ref24] Mao J. (2018). Analysis of genetic diversity and genetic structure of ancient tea trees in Lincang, Yunnan (China): Chinese Academy of Agricultural Sciences.

[ref25] McKenna A. Hanna M. Banks E. Sivachenko A. Cibulskis K. Kernytsky A.. (2010). The genome analysis toolkit: a MapReduce framework for analyzing next-generation DNA sequencing data. Genome Res. 20, 1297–1303. doi: 10.1101/gr.107524.110, PMID: 20644199 PMC2928508

[ref26] Mondal T. K. Bhattacharya A. Laxmikumaran M. Ahuja P. S. (2004). Recent advances of tea (Camellia sinensis) biotechnology. Plant Cell Tiss. Org. Cult. 76, 195–254. doi: 10.1023/B:TICU.0000009254.87882.71

[ref27] Ochanda S. O. Wanyoko J. K. Ruto H. K. (2015). Effect of spices on consumer acceptability of purple tea (Camellia sinensis). Food Nutr. Sci. *06*, 703–711. doi: 10.4236/fns.2015.68073

[ref28] Pickrell J. K. Pritchard J. K. (2012). Inference of population splits and mixtures from genome-wide allele frequency data. PLoS Genetics. 8:e1002967 doi: 10.1371/journal.pgen.1002967 23166502 PMC3499260

[ref29] Purcell S. Neale B. Todd-Brown K. Thomas L. Ferreira M. A. Bender D.. (2007). PLINK: a tool set for whole-genome association and population-based linkage analyses. Am. J. Hum. Genet. 81, 559–575. doi: 10.1086/519795, PMID: 17701901 PMC1950838

[ref30] Ren G. Zhang X. Li Y. Ridout K. Serrano-Serrano M. L. Yang Y.. (2021). Large-scale whole-genome resequencing unravels the domestication history of Cannabis sativa. Sci. Adv. 7:eabg2286. doi: 10.1126/sciadv.abg2286, PMID: 34272249 PMC8284894

[ref31] Schiffels S. Durbin R. (2014). Inferring human population size and separation history from multiple genome sequences. Nat. Genet. 46, 919–925. doi: 10.1038/ng.3015, PMID: 24952747 PMC4116295

[ref32] Slatkin M. (2008). Linkage disequilibrium—understanding the evolutionary past and mapping the medical future. Nat. Rev. Genet. 9, 477–485. doi: 10.1038/nrg2361, PMID: 18427557 PMC5124487

[ref33] Wang X. Feng H. Chang Y. Ma C. Wang L. Hao X.. (2020). Population sequencing enhances understanding of tea plant evolution. Nat. Commun. 11, 1–10. doi: 10.1038/s41467-020-18228-8 32895382 PMC7477583

[ref34] Wang K. Li M. Hakonarson H. (2010). ANNOVAR: functional annotation of genetic variants from high-throughput sequencing data. Nucleic Acids Res. 38:e164. doi: 10.1093/nar/gkq603, PMID: 20601685 PMC2938201

[ref35] Wei C. Yang H. Wang S. Zhao J. Liu C. Gao L.. (2018). Draft genome sequence of Camellia sinensis var. sinensis provides insights into the evolution of the tea genome and tea quality. Proc. Natl. Acad. Sci. 115, E4151–E4158. doi: 10.1073/pnas.1719622115 29678829 PMC5939082

[ref36] Weir B. S. Hill W. G. (2002). Estimating F-statistics. Annu. Rev. Genet. 36, 721–750. doi: 10.1146/annurev.genet.36.050802.093940, PMID: 12359738

[ref37] Willson K.C. Clifford M.N. (2012). Tea: Cultivation to Consumption. Berlin/Heidelberg: Springer Science & Business Media.

[ref38] Xia E. Tong W. Hou Y. An Y. Chen L. Wu Q.. (2020). The reference genome of tea plant and resequencing of 81 diverse accessions provide insights into its genome evolution and adaptation. Mol. Plant 13, 1013–1026. doi: 10.1016/j.molp.2020.04.010, PMID: 32353625

[ref39] Xia E.-H. Zhang H.-B. Sheng J. Li K. Zhang Q.-J. Kim C.. (2017). The tea tree genome provides insights into tea flavor and independent evolution of caffeine biosynthesis. Mol. Plant 10, 866–877. doi: 10.1016/j.molp.2017.04.002, PMID: 28473262

[ref40] Yang J. Lee S. H. Goddard M. E. Visscher P. M. (2011). GCTA: a tool for genome-wide complex trait analysis. Am. J. Hum. Genet. 88, 76–82. doi: 10.1016/j.ajhg.2010.11.011, PMID: 21167468 PMC3014363

[ref41] Zhang C. Dong S.-S. Xu J.-Y. He W.-M. Yang T.-L. (2019). PopLDdecay: a fast and effective tool for linkage disequilibrium decay analysis based on variant call format files. Bioinformatics 35, 1786–1788. doi: 10.1093/bioinformatics/bty875, PMID: 30321304

[ref42] Zhang Q.-J. Li W. Li K. Nan H. Shi C. Zhang Y.. (2020a). The chromosome-level reference genome of tea tree unveils recent bursts of non-autonomous LTR retrotransposons in driving genome size evolution. Mol. Plant 13, 935–938. doi: 10.1016/j.molp.2020.04.009, PMID: 32353626

[ref43] Zhang W. Rong J. Wei C. Gao L. Chen J. (2018). Domestication origin and spread of cultivated tea plants. Biodivers. Sci. 26, 357–372. doi: 10.17520/biods.2018006

[ref44] Zhang W. Zhang Y. Qiu H. Guo Y. Wan H. Zhang X.. (2020b). Genome assembly of wild tea tree DASZ reveals pedigree and selection history of tea varieties. Nat. Commun. 11, 1–12. doi: 10.1038/s41467-020-17498-6 32709943 PMC7381669

[ref45] Zhao H. Sun S. Ding Y. Wang Y. Yue X. Du X.. (2021). Analysis of 427 genomes reveals moso bamboo population structure and genetic basis of property traits. Nat. Commun. 12, 1–12. doi: 10.1038/s41467-021-25795-x 34526499 PMC8443721

[ref46] Zhao D.-W. Yang J.-B. Yang S.-X. Kato K. Luo J.-P. (2014). Genetic diversity and domestication origin of tea plant Camellia taliensis (Theaceae) as revealed by microsatellite markers. BMC Plant Biol. 14, 1–12. doi: 10.1186/1471-2229-14-14 24405939 PMC3890520

[ref47] Zhou J. -G. Zhu Y.-X. (2007). Introduction to Tea Science. China Chinese Medicine Press: China Chinese Medicine Press., PMID:

[ref48] Zhou Z. Jiang Y. Wang Z. Gou Z. Lyu J. Li W.. (2015). Resequencing 302 wild and cultivated accessions identifies genes related to domestication and improvement in soybean. Nat. Biotechnol. 33, 408–414. doi: 10.1038/nbt.3096, PMID: 25643055

