# Supplementary material for: Whole-genome resequencing reveals the origin of tea in Lincang
Source: Front Plant Sci. 2022 Sep 15;13:984422. doi: 10.3389/fpls.2022.984422 (PMC9520660; doi:10.3389/fpls.2022.984422)
Supplement: Supplementary file 2 [file Data_Sheet_1.docx]

Supplementary Material

Supplementary Figures and Tables

Supplementary Figures

**
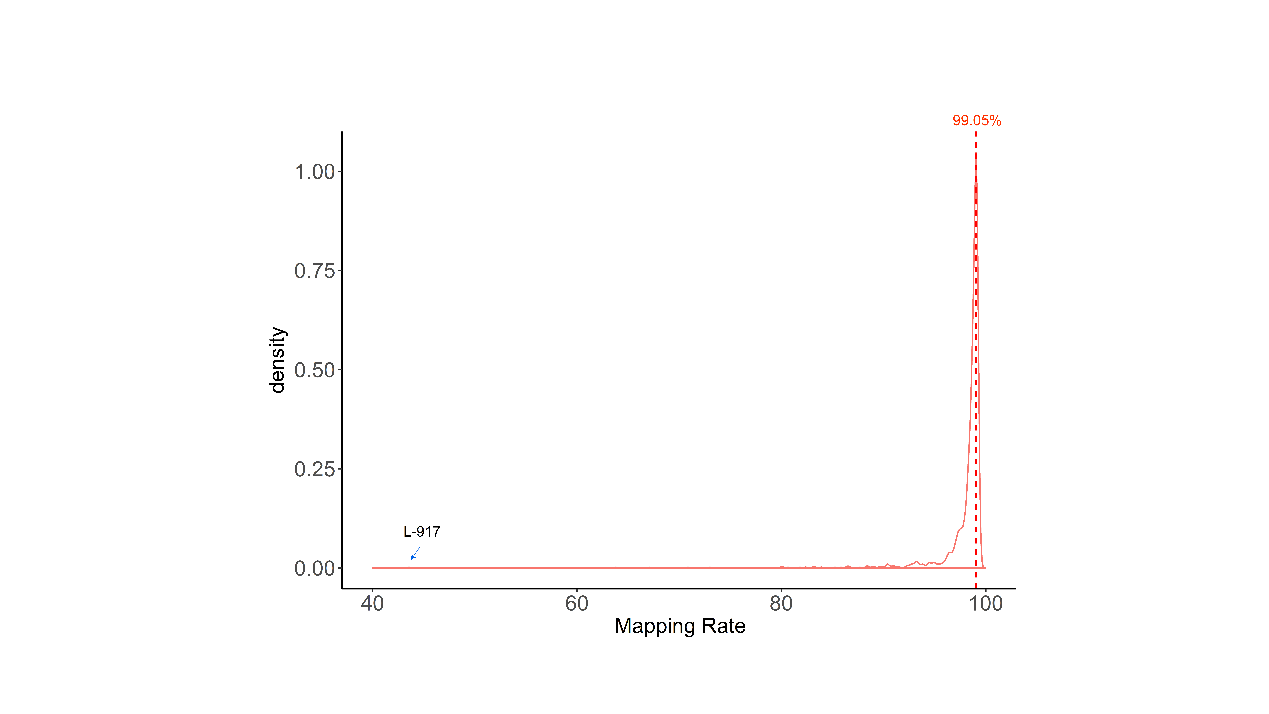
** **Supplementary Figure 1.** The density plot of the sample's mapping rate. The x-coordinates indicate the mapping rate and the y-coordinates indicate the density.

a

c

b

d

e

f

**Supplementary Figure 2.** Density distribution plot of SNPs hard filter

**­­­­­**

a

b

c

d

e

**Supplementary Figure 3.** Density distribution plot of indels hard filter

**
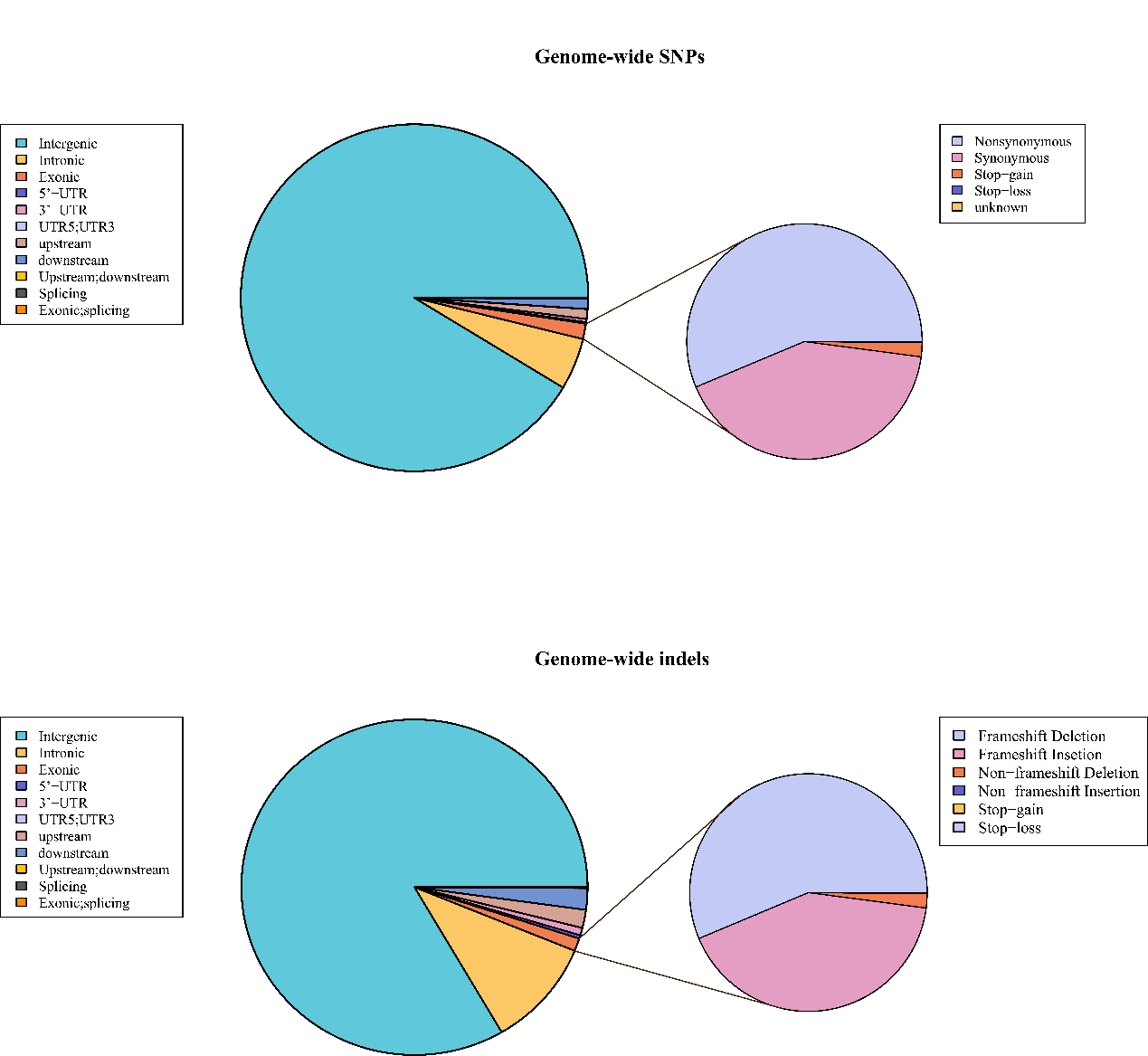
**

a

b

**Supplementary Figure 4.** Whole-genome SNPs and indels distribution are shown in table 2 and table 3.

**
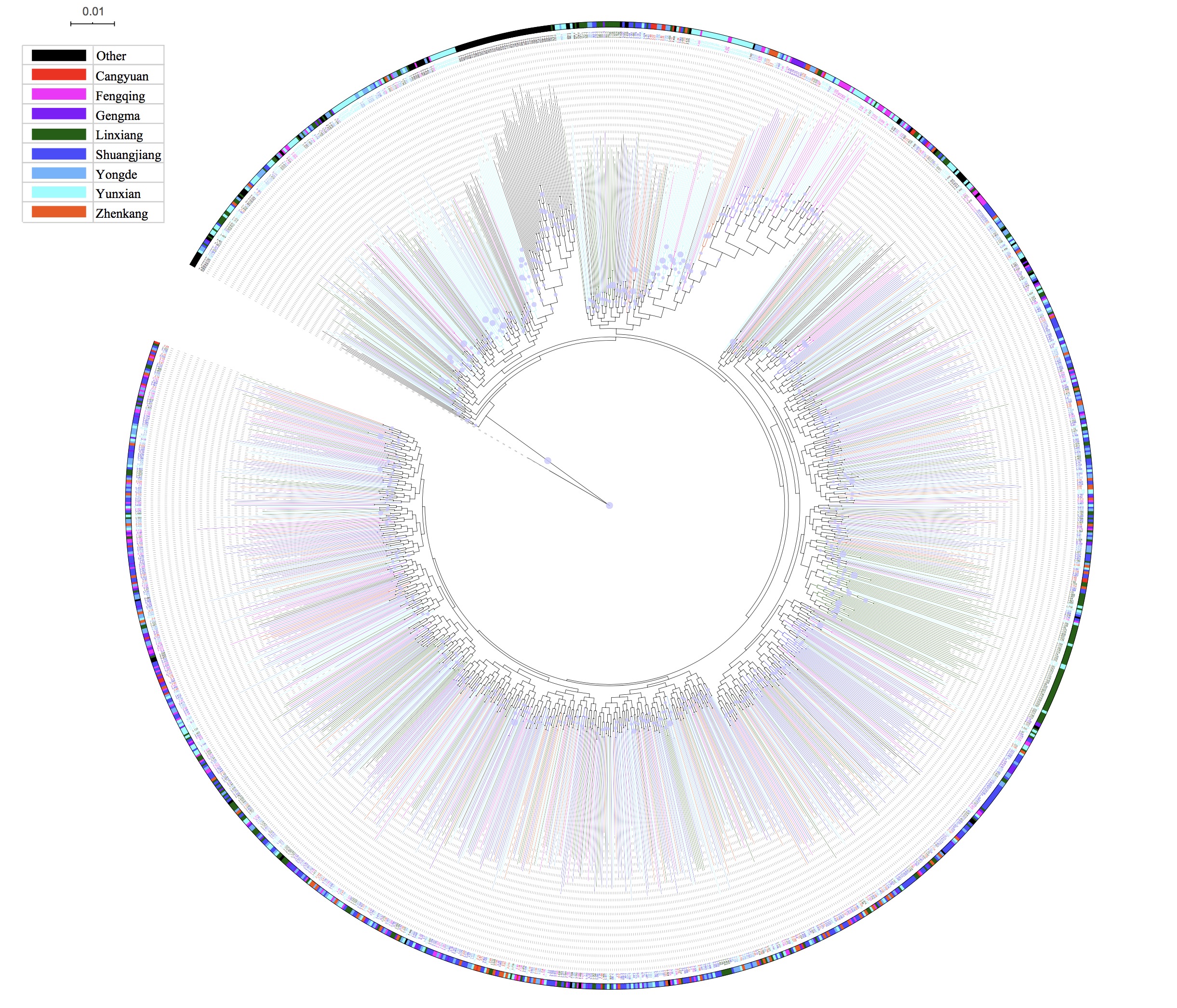
**

**Supplementary Figure 5.** Phylogenetic relationships of 1350 tea plants from nine different regions. KM6 (*C. cuspidata*) was selected as the outgroup.


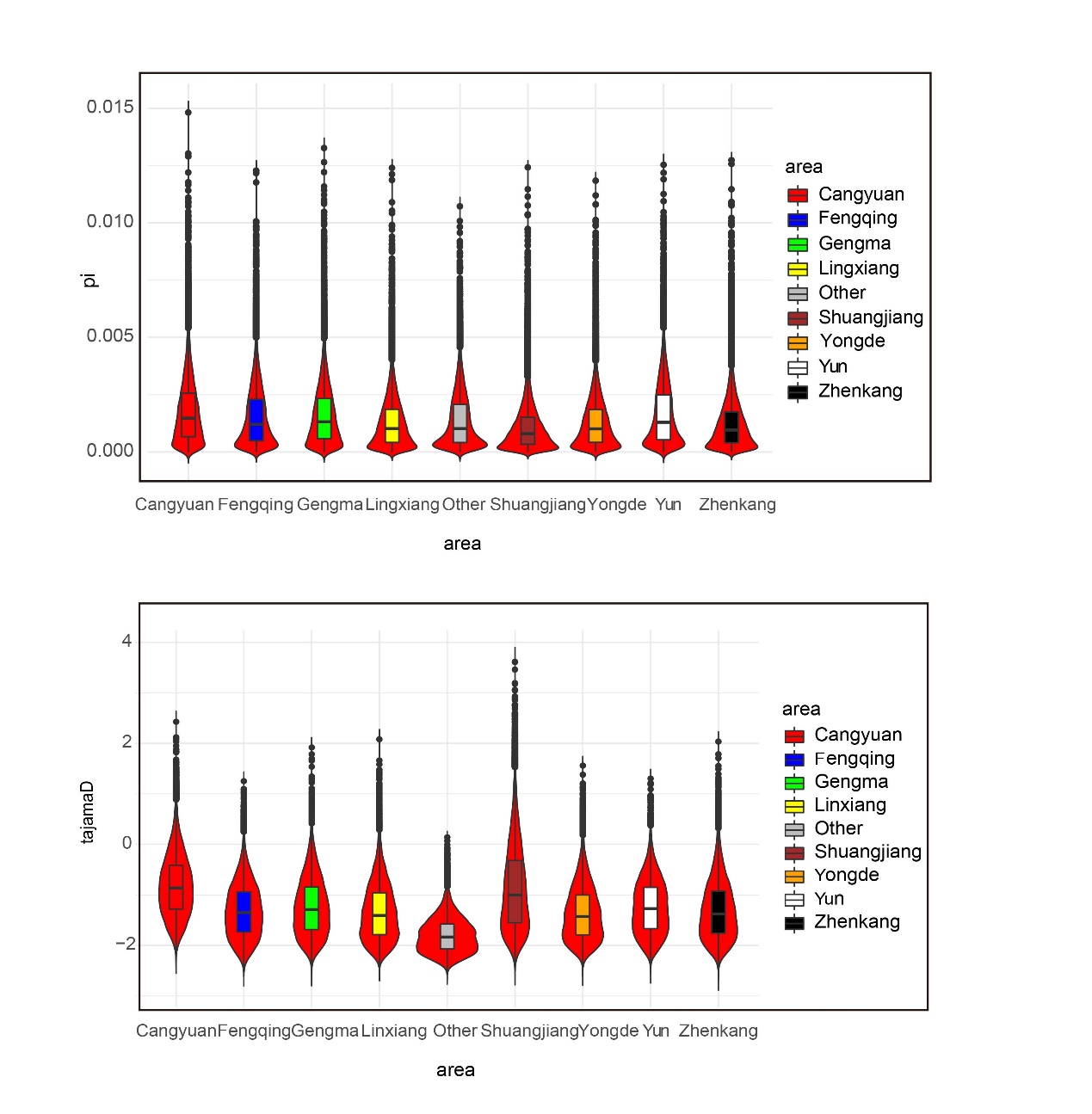
**Supplementary Figure 6.** (a) Nucleotide diversity (*θ*_π_) estimation of the tea plant from different geographic regions. (b) Tajima’*D* estimation of the tea plant from different geographic regions.

a

b

**
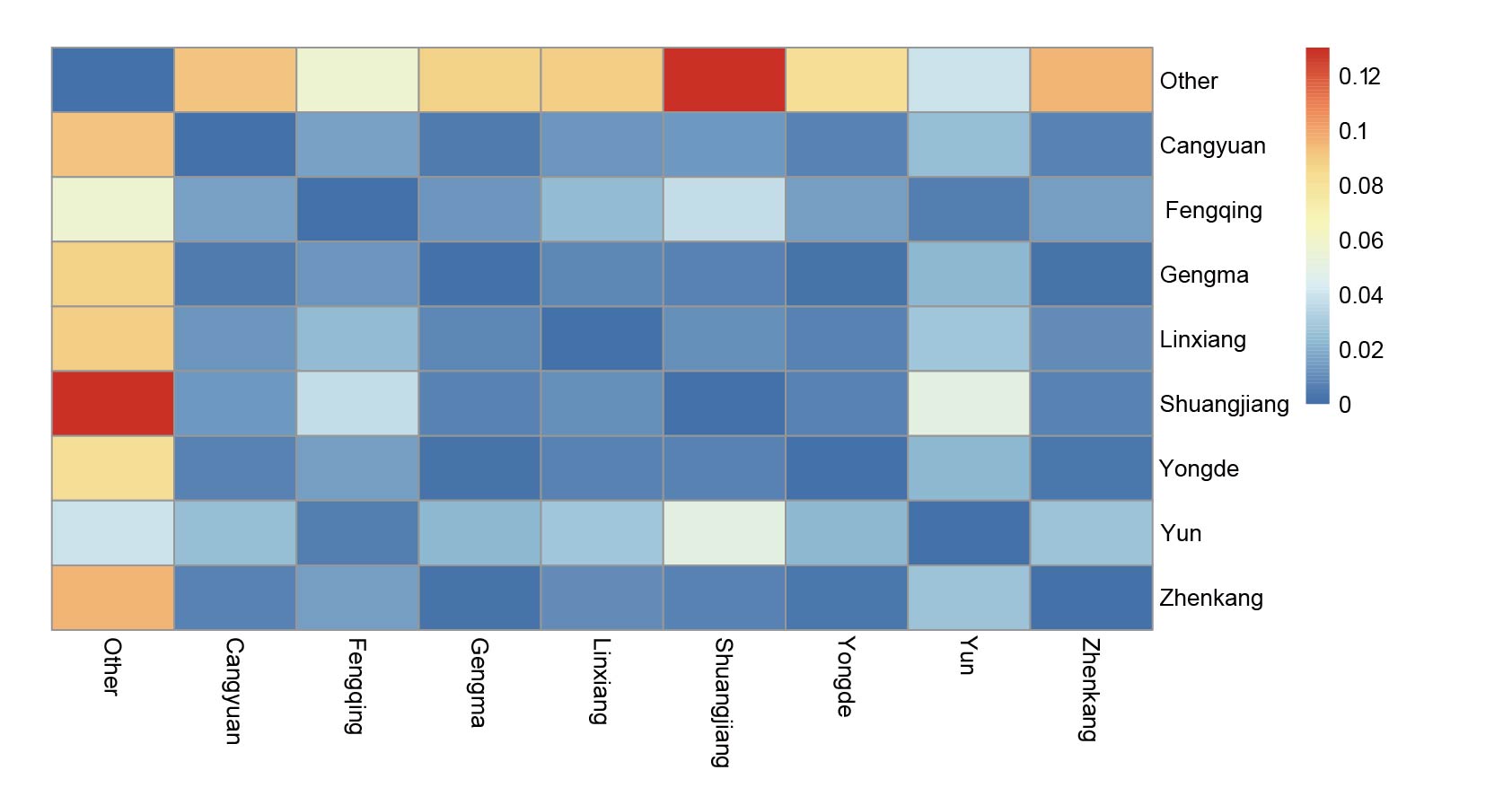
**

**Supplementary Figure 7.** Genetic differentiation (*F_ST_*) within different area populations. The color marks between each regional population indicate the Weir and Cockerham weighted Fst values.


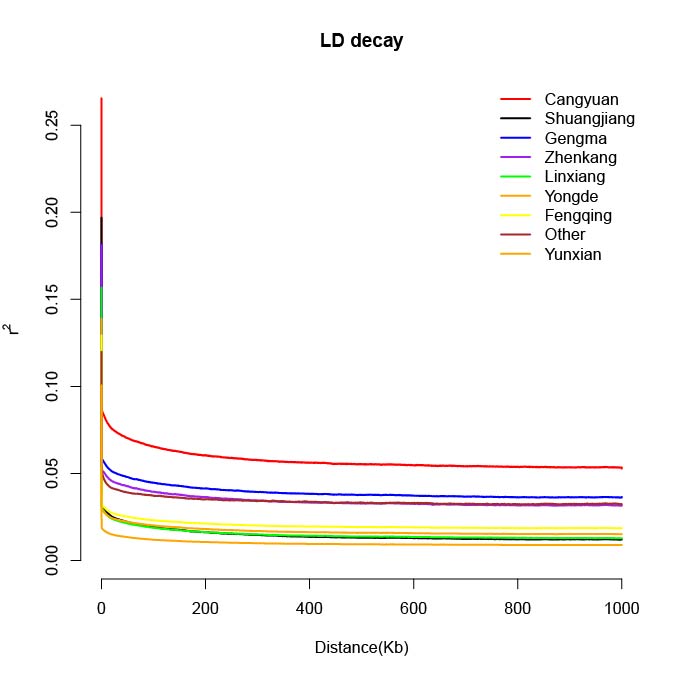


**Supplementary Figure 8.** LD decay in different regional populations. The x-coordinates indicate the distance between bases and the y-coordinates indicate the mean value of the correlation coefficient.**
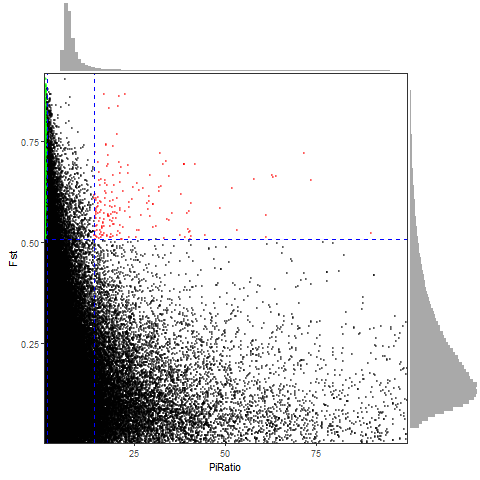
**

**Supplementary Figure 9.** Selective sweep regions during domestication inferred from FST and π statistics of group1 and group2.


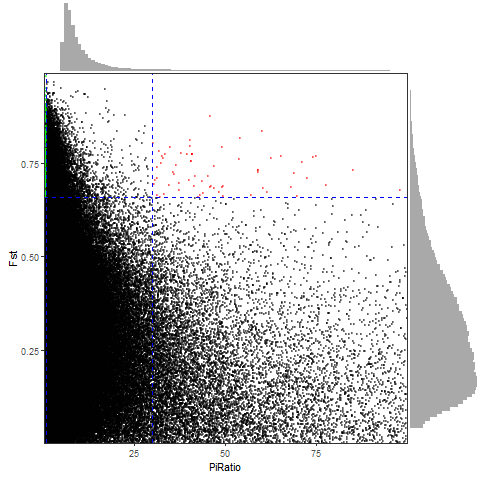


**Supplementary Figure 10.** Selective sweep regions during domestication inferred from FST and π statistics of group1 and group3.

**
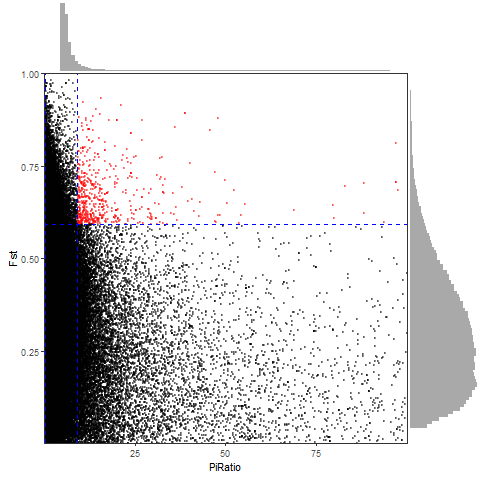
**

**Supplementary Figure 11.** Selective sweep regions during domestication inferred from FST and π statistics of group2 and group3.


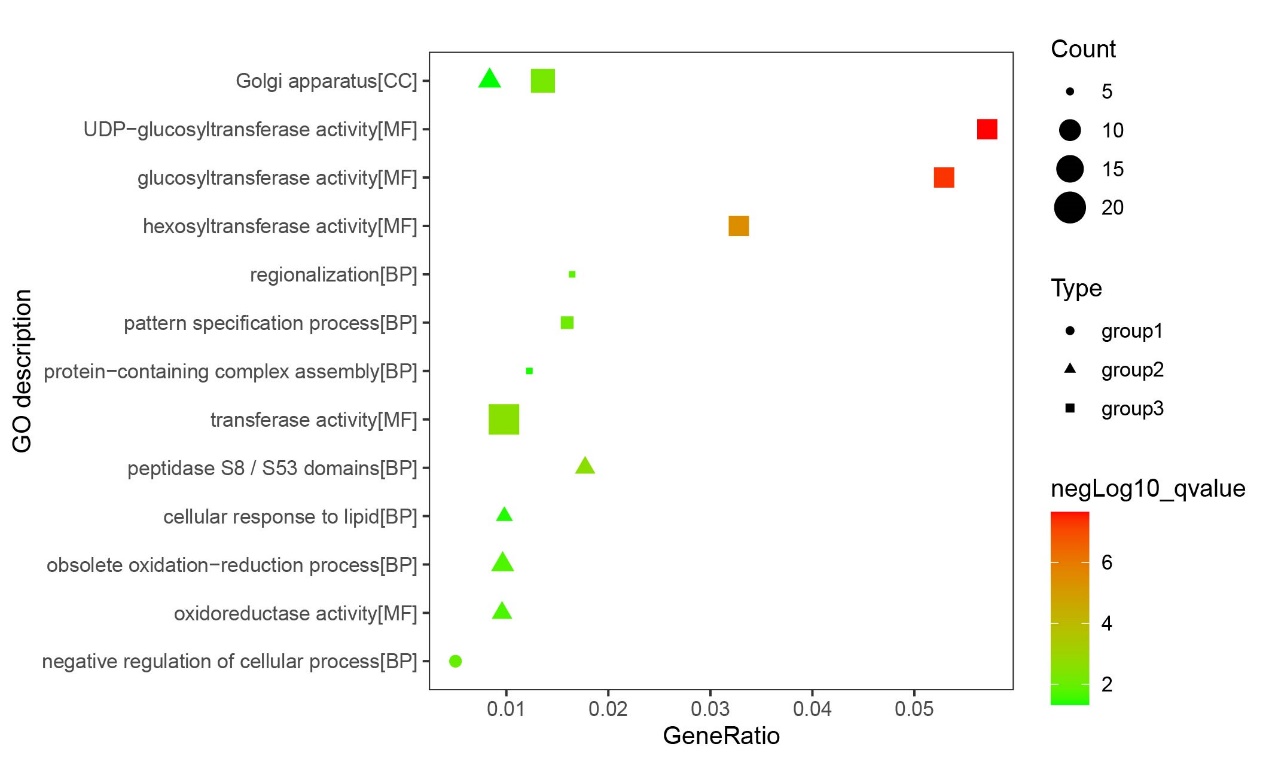


**Supplementary Figure 12**. GO enrichment of the candidate selective sweep genes. The color of the bubble shows the corresponding q-value. The shape of the bubble shows the different subgroup.GeneRatio shows the percentage of enriched genes out of the total number in the GO category.
